# The complete mitochondrial genome of the gadwall (*Anas strepera*)

**DOI:** 10.1080/23802359.2019.1667277

**Published:** 2019-09-20

**Authors:** Qinguo Wei, Xiaoyang Wu, Yanran Hu, Guolei Sun, Jun Chen, Xiaodong Gao, Weilai Sha, Honghai Zhang

**Affiliations:** a Jiangsu Key Laboratory for Biodiversity and Biotechnology, College of Life Sciences, Nanjing Normal University, Nanjing, Jiangsu, China;; b College of Life Sciences, Qufu Normal University, Qufu, Shandong, China;; c School of Life Sciences, Central South University, Hunan, China;; d College of Marine Life Sciences, Ocean University of China, Qingdao, Shandong, China

**Keywords:** Anatidae, *Anas strepera*, mitogenome

## Abstract

The gadwall *Anas strepera* was widely distributed migratory duck in the family of Anatidae. The complete mitochondrial genome of gadwall was sequenced in this study to explore the mitogenomic characteristics and figure out its phylogenetic relationships within Anatidae. The mitogenome is a circular DNA molecule of 16600 bp in length with 13 protein-coding genes, 22 tRNA genes, 2 rRNA genes and a control region. The overall base composition of the mitogenome was A: 28.84%, T: 22.19%, G: 16.15%, C: 32.81%. Phylogenetic analysis showed that the *Anas strepera* was closed to *Anas platyrhynchos*.

The gadwalls *Anas strepera* are medium-sized dabbling duck which distributed widely in many areas of the world. It has been listed as Least Concern conservation status by IUCN (BirdLife International 2016). This species prefer to inhabit in sheltered, shallow, standing or slow-flowing waters with ample vegetation and grass-covered islands that can provide cover for nesting (Greenwood et al. 1995; Kear 2005). Previous studies mainly focused on its habitat selection and systematic geography (Peters et al. 2008; Shaffer et al. 2011). Nevertheless, the mitogenome character of *Anas strepera* is still unclear. In this study, the complete mitogenome of *Anas strepera* were sequenced, which could provide useful genetic resources for the future study of this species.

The muscle tissue was obtained from an accident dead individual in Nansi lake Nature Reserve, Shandong, China (35°19′725″N, 116°36′937″E). The specimen was stored in the Animal Specimen Museum of Qufu Normal University, Qufu, Shandong, China. And the accession number is QFA20150036. We amplified the mitogenome with 15 pairs of primers and sequenced with ABI 3730 sequencer. The obtained sequences were aligned and assembled with the software MEGAX (Kumar et al. 2018). The mitogenome of the *Anas strepera* is 16600 bp in length and had been deposited in GenBank (MN186586). Character analysis showed that the genome contained 13 protein-coding genes, 2 rRNA genes, 22 tRNA genes and 1 control region, in which, 28 genes are encoded on the heavy (H) strand and the remaining 9 genes are encoded on light (L) strand, including ND6 and 8 tRNA genes. The base composition of the sequence was A: 28.84%, T: 22.19%, G: 16.15%, C: 32.81%.

Phylogenetic relationships of 13 Anatidae bird including *Anas strepera* were analyzed with the maximum likelihood (ML) and the Bayesian inference (BI) methods based on their 13 mitochondrial protein-coding genes, with *Phasianus colchicums* (NC_015526.1) as an outgroup. The trees obtained from the two methods shared the same topology, with strong support for all nodes (Figure 1). The phylogenetic analysis results showed that the *Anas strepera* was closed to *Anas platyrhynchos.*

**Figure 1.**
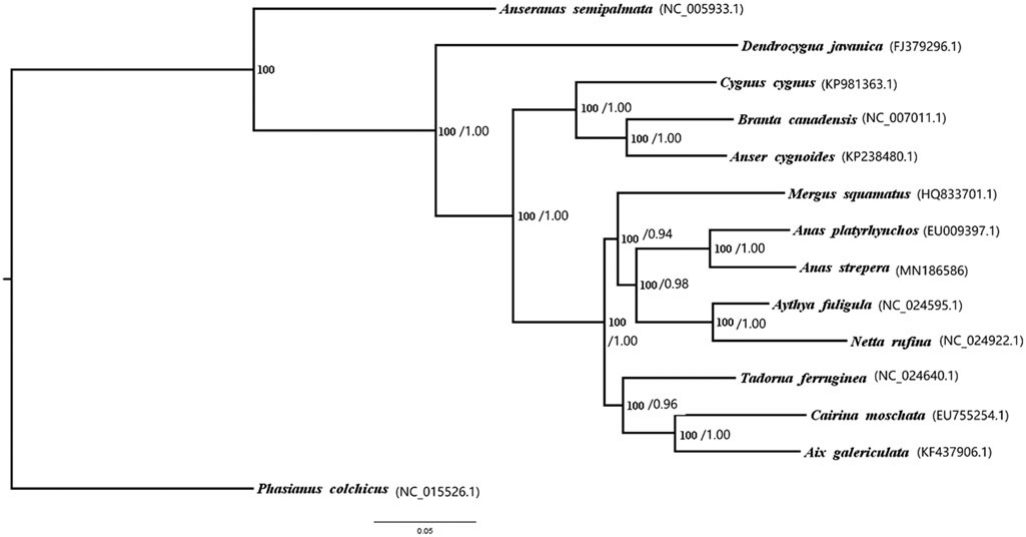
Maximum likelihood (ML) and Bayesian phylogenetic inference (BI) trees of 13 Anatidae species based on 13 protein-coding genes. BI posterior probabilities/ML bootstrap values are shown behind the nodes. GenBank accession numbers for each species are shown in parentheses.
